# Shunt Dysfunction Assessment in Shunted Patients via Multiparametric Non-Invasive ICP Waveform Monitoring

**DOI:** 10.3390/biomedicines13102436

**Published:** 2025-10-07

**Authors:** Raphael Bertani, Gabriel Semione, Milene Zanella Capitanio, Sérgio Brasil, Sávio Batista, Gabriel André da Silva Mendes, Caio Perret, Christian Ferreira, Wellingson Silva Paiva, Fernando Campos Gomes Pinto

**Affiliations:** 1 Division of Neurosurgery, Department of Neurology, University of São Paulo, São Paulo 05508-220, Brazil; sbrasil@alumni.usp.br (S.B.); wellingson.paiva@hc.fm.usp.br (W.S.P.); neurofernando@gmail.com (F.C.G.P.); 2 Faculty of Medicine, University of West Santa Catarina, Joaçaba 89600-000, Brazil; gabrieldasilvasemione@gmail.com; 3 Faculty of Medicine, Community University of Chapecó Region, Chapecó 89809-900, Brazil; milenezcapitanio@gmail.com; 4 Department of Neurology, Emory University, Atlanta, GA 30322, USA; saviobatista360@gmail.com; 5 São Paulo State Public Servant’s Hospital, São Paulo 04024-002, Brazil; mendes1986@gmail.com; 6 Biomedical Sciences Institute, Federal University of Rio de Janeiro, Rio de Janeiro 21941-853, Brazil; perretcaio@gmail.com; 7 Department of Neurosurgery, Phelps Hospital/Northwell Health, Sleepy Hollow, NY 10591, USA; cferreira6@northwell.edu

**Keywords:** non-invasive ICP waveform, ventriculoperitoneal shunt, shunt dysfunction, intracranial compliance, hydrocephalus

## Abstract

**Background/Objectives**: Ventriculoperitoneal shunts are the standard treatment for adults diagnosed with several CSF disorders, but often face dysfunction, leading interest in non-invasive methods for diagnosing shunt issues. This study evaluates the potential of non-invasive intracranial pressure waveform monitoring (nICPw) with the brain4care (B4C) system to distinguish overdrainage, underdrainage, and normal shunt function in patients with CSF disorders. **Methods**: In this single-center, observational study at Hospital das Clínicas, Brazil, adult patients with CSF shunts were enrolled. Patients were categorized as Overdrainage or Underdrainage, based on clinical parameters, with an Asymptomatic group. The B4C system provided nICPw monitoring, and six parameters (including various P2/P1 ratios) were analyzed via MANOVA and ANOVA. **Results**: Among 30 patients (6 overdrainage, 6 underdrainage, 18 asymptomatic), five asymptomatic patients were excluded from the main analysis due to incomplete data collection, leaving 25 patients. Overdrainage patients had significantly higher ΔP2/P1 values (0.618 ± 0.210) than asymptomatic ones (0.227 ± 0.171). After excluding outliers, differences were more pronounced (H = 10.89; *p* < 0.01). Underdrainage patients had intermediate ΔP2/P1 values (0.387 ± 0.179) and consistently higher P2/P1 averages (>1.3). ROC analysis indicated that ΔP2/P1 > 0.3 suggested shunt dysfunction (AUC = 0.731), while the highest P2/P1 offered stronger discrimination (AUC = 0.782). A global average P2/P1 > 1.3 was linked to underdrainage, with the lowest P2/P1 values differentiating overdrainage (0.948 ± 0.321) from underdrainage (1.143 ± 0.156). **Conclusions**: nICPw monitoring with the B4C system demonstrated potential for detecting shunt dysfunction. Combining parameters, especially ΔP2/P1 and highest P2/P1, improves diagnostic accuracy, offering a non-invasive method that may aid in distinguishing normal from abnormal shunt function.

## 1. Introduction

Hydrocephalus is a neurological condition characterized by enlarged cerebral ventricles (ventriculomegaly) resulting from excessive cerebrospinal fluid (CSF) accumulation in the ventricles and subarachnoid spaces of the brain [1]. This abnormal accumulation of CSF can lead to increased intracranial pressure (ICP) and reduced intracranial compliance (ICC). If left untreated, impaired ICC leads to a range of severe neurological complications [1], potentially compromising the patient’s quality of life.

The preferred treatment for hydrocephalus in adults is the surgical implantation of a ventriculoperitoneal shunt (VPS) [2]. This procedure involves the insertion of a catheter into the cerebral ventricle, redirecting CSF to the peritoneal cavity, where it is reabsorbed. VPS has been shown to alleviate symptoms in many patients effectively; however, shunt dysfunction remains a significant and recurrent complication that can lead to the recurrence of symptoms [3], with shunt underdrainage occurring in approximately 17–22.7% of cases [4,5]. Such dysfunctions can result in serious neurological consequences [1,2], often necessitating revision surgery, which is widely regarded as the gold standard for managing these complications [6].

Early detection of shunt dysfunction is crucial for preventing severe complications. However, some methods used for shunt dysfunction diagnosis, such as computed tomography (CT) and magnetic resonance imaging (MRI), have significant limitations [7]. Besides being costly and often inaccessible for continuous patient follow-up [6], these imaging modalities provide only a snapshot of the patient’s condition and may be incomplete or nonspecific due to the lack of a dynamic view, making diagnosis challenging for clinicians [1,6]. Given these limitations, there is a growing demand for alternative monitoring methods capable of providing real-time data for shunt dysfunction diagnosis based on patient intracranial dynamics.

An FDA-cleared (K240821) [8] non-invasive ICP monitoring device, the brain4care (B4C) System, has emerged as a promising tool for real-time monitoring of intracranial dynamics, offering continuous assessment of ICP variations and ICC. The system detects micrometric pulsatile cranial expansions resulting from ICP fluctuations and non-invasively reproduces ICP waveforms [9,10]. By analyzing the morphology of these surrogate waveforms and derived parameters, such as the P2/P1 ratio, which reflects the relative amplitude of the tidal wave (P2) to the percussion wave (P1) [11], the device provides a non-invasive means to monitor ICC [10].

In this context, the objective of this study was to evaluate the feasibility and clinical applicability of the B4C System’s non-invasive P2/P1 ratio in detecting shunt dysfunction, such as overdrainage, underdrainage, and normal shunt function, in patients with hydrocephalus, particularly in the context of suspected impaired ICC.

## 2. Materials and Methods

### 2.1. Study Design

An observational single-center study was executed at Hospital das Clínicas, University of São Paulo, Brazil, with the aim to evaluate the feasibility and applicability of a non-invasive technique of intracranial dynamics monitoring for diagnosing shunt dysfunction. Patients with suspected shunt dysfunction were compared with patients with no complaints, i.e., stable and with no suspicion of shunt dysfunction. All patients had CSF shunts.

### 2.2. Participants

The study included shunted adults (>18 y.o.) with diagnosed cerebral hydrodynamic disorders requiring shunts, such as obstructive or communicating hydrocephalus, intermittent pressure hydrocephalus, benign intracranial hypertension, and other CSF circulation disorders. The exclusion criteria were patients under 18 years of age and those who did not provide informed consent. Patients were included consecutively, as they were seen in clinic. Patients that did not agree to participate in the study or were found to have missing data were excluded.

Symptomatic patients were divided into two groups based on clinical suspicions observed during physical examination and analysis of clinical history. One group was identified as “Overdrainage” and the other “Underdrainage”.

Overdrainage patients, characterized by excessive CSF drainage [12], were identified as those presenting headaches that improved when lying down and worsened when sitting or standing up, with or without nausea [13,14].

Underdrainage patients, characterized by insufficient CSF drainage [15], were identified as those having headaches that did not improve with positional changes or those that had improvements when standing or sitting up, with or without nausea. The underdrainage symptoms had to be persistent after 2 consultations with at least a 30-day interval or if the patient was submitted to surgery and experienced relief of symptoms. A complete list of symptoms can be found on Table S1.

When combined, both groups were referred to as the “Shunt dysfunction” group. A third group composed of stable shunted patients with no symptoms of underdrainage or overdrainage was defined as the “Asymptomatic” group. The inclusion in each group was exclusively based on clinical parameters. Despite having been diagnosed with shunt dysfunction, not all patients were subjected to revision surgeries.

Asymptomatic patients had no complaints and were being followed for prevention and continued observation, the data are shown in Table S2. Shunt dysfunction patients had symptoms and expressed straightforward complaints during clinic visits or were admitted via the emergency department.

The study initially recruited 35 shunted adult patients. However, 5 patients were excluded from the main analysis due to incomplete data collection (patients who did not have monitoring sessions in all required positions). Therefore, the final analysis included 30 patients: 18 Asymptomatic, 6 Overdrainage, and 6 Underdrainage.

### 2.3. Ethical Considerations

The study protocol was reviewed and approved by the Faculty of Medicine Ethics Committee of the Hospital das Clínicas University of São Paulo (under CAAE number 76451323.4.1001.0068), following the Declaration of Helsinki guidelines. All participants or their legal representatives provided written informed consent before enrollment.

### 2.4. Monitoring Procedure

The non-invasive ICP waveform (nICPw) was monitored using the B4C System. Data was recorded and visualized using the brain4care mobile app [16].

The monitoring sessions lasted 15 min, with patients placed in three different positions: lying down, bedrest at 30°, and standing up. These positions were used to assess the effect of postural changes on ICP waveforms. The positional changes were designed to test the patients’ intracranial compensatory reserve and how they were reflected as variations in the B4C System’s device output (variations on estimated P2/P1 ratio). The collected data was processed with the B4C System’s analytical engine that derive clinical parameters, including the estimated P2/P1 ratio, revealing ICP changes [8] and the ICC status [17]. It is worth noting that, while waveform acquisition is a simple process, basic training is required in order for waveforms to be reproducible. The brain4care company provides training to all users before initiating research projects or clinical use.

### 2.5. Statistical Analysis

This investigation employed a statistical framework to evaluate the discriminatory capacity of various clinical parameters in distinguishing between patients with shunt dysfunction (categorized as Overdrainage or Underdrainage) and those who remained Asymptomatic. The clinical parameters were defined as: ΔP2/P1 (the difference between the lowest and highest P2/P1 values during the full 15 min monitoring session), Average 30° P2/P1 (average ratio in the 30° position), Average Lying P2/P1 (average ratio in the lying position), Average Standing P2/P1 (average ratio in the standing position), and Lowest P2/P1 (the lowest observed P2/P1 ratio) and Highest P2/P1 (the highest observed P2/P1 ratio). The methodological approach progressed systematically from etiological diagnosis analysis utilizing Multivariate Analysis of Variance (MANOVA) and Analysis of Variance (ANOVA) tests on single parameter analysis of ΔP2/P1 values across patient groups via non-parametric statistical methods. Subsequently, a more sophisticated combined parameters analysis was implemented through MANOVA and Linear Discriminant Analysis to identify the principal factors driving group differentiation. The analysis culminates in an assessment of predictive capability through a combined model incorporating key metrics (ΔP2/P1, Highest P2/P1, and Average Standing P2/P1), evaluated via logistic regression with stratified cross-validation protocols and comprehensive receiver operating characteristic (ROC) curve analysis to quantify discriminative performance across multiple clinical scenarios.

### 2.6. Power Analysis

To ensure adequate sample size, we performed power analysis based on our preliminary dataset. The aim was to assess sample size adequacy for the purpose of executing an analysis that will guide future studies.

### 2.7. Etiological Diagnosis Analysis

To verify statistical differences between each pathology, patients were grouped by etiological diagnosis and compared across all parameters: average P2/P1 while lying, 30°, and standing positions as well as the highest, lowest, and ΔP2/P1 values using a MANOVA test. An additional univariate ANOVA test was performed for each individual metric to detect specific group-level differences, and also to explore the influence of diagnoses on key metrics, such as revisions within each group. Statistical significance was defined as *p* < 0.05.

### 2.8. Outlier Considerations

To ensure robustness, we identified and excluded outliers in the Asymptomatic group. Outliers were based on the following criteria, and analyzed separately: asymptomatic patients with ΔP2/P1 values exceeding 0.3 with a diagnosis suggestive of diffuse brain injury mechanisms, such as post-infectious hydrocephalus and post-traumatic hydrocephalus, were excluded. We believe this subgroup of patients, with pathologies related to diffuse neuroinflammation, may behave similarly to patients presenting shunt dysfunction. This subgroup was also thoroughly examined during etiological diagnosis analysis.

### 2.9. Single Parameters Analysis

To verify if ΔP2/P1 values differed significantly between population groups, the analysis focused on the ΔP2/P1 values across the three groups. To allow a proper comparison of the aforementioned groups, we used the Kruskal–Wallis test, a non-parametric alternative to ANOVA that does not assume normality. This test compared all groups, however it did not indicate where exactly the statistical significance was, if found. Therefore, we also performed pairwise comparisons using the Wilcoxon rank-sum test with the Holm test. With these, we identified which comparisons (e.g., asymptomatic vs. overdrainage, overdrainage vs. underdrainage, etc.) had statistical significance.

### 2.10. Combined Parameter Analysis

To evaluate the diagnostic potential of waveform-derived metrics across patient groups, we conducted a series of multivariate analyses. Initially, we performed a Multivariate Analysis of Variance (MANOVA) to determine whether the three groups—Asymptomatic, Overdrainage, and Underdrainage—differed significantly across six parameters: ΔP2/P1, Average 30° P2/P1, Average Lying P2/P1, Average Standing P2/P1, Lowest P2/P1, and Highest P2/P1. A 95% confidence threshold (*p* < 0.05) was used to assess statistical significance.

To further investigate group separation, we applied Linear Discriminant Analysis (LDA) using all six metrics. Canonical variates were derived to maximize inter-group separation, and variable contributions were assessed via discriminant coefficients. Assumptions of normality, homogeneity of variance–covariance matrices, and multicollinearity were evaluated to ensure model robustness. LDA model accuracy was assessed through cross-validation, and the discriminant functions were interpreted to identify the most influential features for class differentiation.

### 2.11. ROC Curve and Combined Model Evaluation

For predictive classification, we implemented logistic regression models to evaluate how well combined waveform features distinguished between clinical groups. We constructed multiple binary classifiers targeting the following comparisons: (i) Shunt Dysfunction (Overdrainage + Underdrainage) vs. Asymptomatic, (ii) Underdrainage vs. Asymptomatic, (iii) Overdrainage vs. Asymptomatic, and (iv) Overdrainage vs. Underdrainage. Each model included three pre-selected parameters to avoid multicollinearity.

To mitigate overfitting in this small-sample setting, we used stratified 5-fold cross-validation. In each fold, the dataset was split into five groups of six patients; models were trained on four folds (n = 24) and tested on the remaining fold (n = 6). This procedure was repeated five times so that each group served as a test set once.

To further quantify variability in model performance, we employed bootstrap resampling with 1000 iterations, generating distributions of the area under the ROC curve (AUC) for each binary comparison. Mean AUC and standard deviation (reported as AUC ± SD) were calculated, allowing us to assess diagnostic stability across comparisons.

### 2.12. Surgical Group Subanalysis

In addition to the main analysis of the complete patient cohort, we planned a specific subanalysis of patients who underwent surgical intervention. This surgical subanalysis evaluated changes in nICPw parameters before and after shunt revision surgery. For the subset of patients who received surgical correction (n = 6), we collected paired pre-operative and post-operative measurements of all nICPw parameters. Due to the limited sample size in this surgical subgroup, patients were not separated into Overdrainage and Underdrainage categories but were analyzed as a combined Shunt dysfunction group. Paired t-tests were used to compare pre-operative and post-operative values for each parameter: ΔP2/P1, Average Lying P2/P1, Average 30° P2/P1, and Average Standing P2/P1. Statistical significance was set at *p* < 0.05.

## 3. Results

Of the 30 patients analyzed, 18 were classified as Asymptomatic, while 12 presented with suspected shunt dysfunction (6 Overdrainage, 6 Underdrainage). Five asymptomatic patients with disproportionately high ΔP2/P1 values and histories of post-infectious or post-traumatic hydrocephalus were identified as outliers and excluded from refined analyses. This adjustment resulted in a revised cohort of 25 patients.

### 3.1. Power Analysis

The power analysis revealed that our sample size (n = 30) was sufficient for detecting large effect sizes observed in this preliminary investigation. Statistical calculations indicated that for detecting medium effect sizes (Cohen’s d ≈ 0.5) with 80% power at an alpha level of 0.05, approximately 64 subjects per group would be optimal for future validation studies. Despite the limitations of our current sample size, the observed large effect sizes provide preliminary evidence supporting the potential diagnostic utility of the measured parameters, while acknowledging the need for cautious interpretation due to wide confidence intervals.

### 3.2. Etiological Diagnosis Analysis

The results indicated no statistically significant differences (*p* > 0.05) between diagnostic groups when analyzing the entire dataset. When analyzing specific status groups separately, we observed significant differences in Average Lying P2/P1 (*p* = 0.0489) and Average 30° P2/P1 (*p* = 0.0269) across diagnoses within the Asymptomatic group. Further examination of the Asymptomatic group identified five patients with significantly elevated ΔP2/P1 values—four with post-infectious hydrocephalus and one with post-traumatic brain injury hydrocephalus.

When comparing these five patients with the remaining asymptomatic patients, we found a significant difference in ΔP2/P1 values (*p* < 0.001). This finding suggests that pathologies involving diffuse neuroinflammation may result in altered ICC metrics even in clinically asymptomatic individuals, although these findings may have limited utility due to small sample size.

### 3.3. Single Parameter Analysis

The single parameter analysis, focused on the difference in ΔP2/P1 values, revealed significant group-level differences (Kruskal–Wallis test, H = 11.04, *p* = 0.004). The Overdrainage group exhibited significantly higher ΔP2/P1 values (mean 0.618, SD = 0.210) than the Asymptomatic group (mean 0.227, SD = 0.171) (*p* = 0.0024). The Asymptomatic group presented P2/P1 ratio differences while lying (*p* = 0.0489) and at 30° (*p* = 0.0269) when compared to the Shunt dysfunction group.

**Figure 1:**
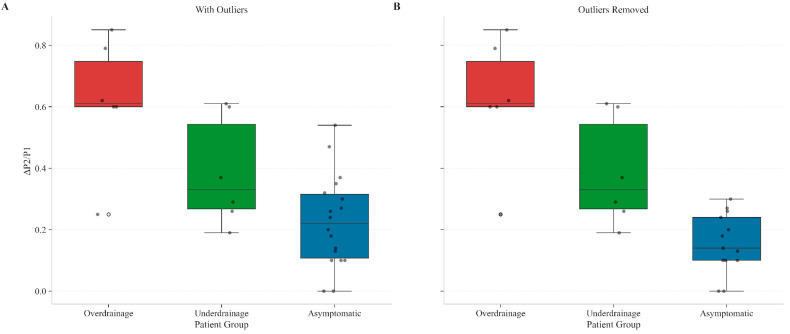
Distribution of ΔP2/P1 Values Across Patient Groups Before and After Outlier Removal. Boxplots showing the distribution of ΔP2/P1 values across patient groups with the full dataset (**A**) and after removing outliers (**B**). In the full dataset (**A**), Asymptomatic (n = 18), Overdrainage (n = 6), and Underdrainage (n = 6) groups are shown. After excluding 5 outliers (**B**), the dataset includes Asymptomatic (n = 13), Overdrainage (n = 6), and Underdrainage (n = 6) patients. Horizontal lines within boxes represent median values, boxes represent interquartile ranges (25th to 75th percentiles), and whiskers extend to the minimum and maximum values within 1.5 times the interquartile range. Individual data points are shown as black dots. In the full dataset (**A**), the Kruskal–Wallis test indicated significant differences among groups (H = 11.04, *p* = 0.004), with Overdrainage exhibiting significantly higher ΔP2/P1 values (mean = 0.611 ± 0.210) than the Asymptomatic group (mean = 0.227 ± 0.171) (*p* = 0.0024). After removing outliers (**B**)—asymptomatic patients with ΔP2/P1 values exceeding 0.3 who had diagnoses of post-infectious and post-TBI hydrocephalus—group differences remained significant (H = 10.89, *p* < 0.01). Pairwise comparisons then revealed significant differences across all groups: Overdrainage vs. Asymptomatic (*p* = 0.0019), Underdrainage vs. Asymptomatic (*p* = 0.0121), and Overdrainage vs. Underdrainage (*p* = 0.0299).

As previously mentioned, asymptomatic patients with ΔP2/P1 values exceeding 0.3 were identified and a subanalysis without them was performed (Figure 1). These patients had diagnoses suggestive of diffuse brain injury mechanisms, including post-infectious hydrocephalus and post-traumatic brain injury (post-TBI) hydrocephalus. Similar analysis of ΔP2/P1 values across groups was executed adjusting for outliers and presented (Kruskal–Wallis test, H = 10.89, *p* < 0.01). Pairwise comparisons using the Wilcoxon test revealed statistically significant differences across all groups. The comparison between Overdrainage and Asymptomatic patients revealed a *p* = 0.0019, whereas the Underdrainage versus Asymptomatic a *p* = 0.0121. Finally, the comparison between Overdrainage and Underdrainage revealed a *p* = 0.0299.

The analysis confirmed that Overdrainage and Underdrainage exhibited distinct ΔP2/P1 alterations compared to the refined Asymptomatic group. The MANOVA results showed a statistically significant difference between groups (Pillai’s Trace = 0.684, *p* = 0.017).

Post hoc pairwise comparisons using Holm’s method revealed that the Asymptomatic vs. Overdrainage analysis revealed a *p* < 0.001, whereas the Overdrainage vs. Underdrainage analysis revealed a *p* = 0.021. Finally, the Asymptomatic vs. Underdrainage analysis had a *p* = 0.067. After reanalyzing without outliers the *p*-values were < 0.001, 0.018 and 0.052, respectively.

Overall, the single parameter analysis demonstrated that ΔP2/P1 may be a powerful discriminator between shunted patient conditions, with distinctly different patterns observed across all three groups. The Overdrainage group showed the highest variability (mean 0.618, SD = 0.210), Underdrainage exhibited intermediate values, and Asymptomatic patients maintained the lowest variability (mean 0.227, SD = 0.171). These statistically significant differences (*p* < 0.01) remained after outlier exclusion.

### 3.4. Combined Parameter Analysis

**Figure 2:**
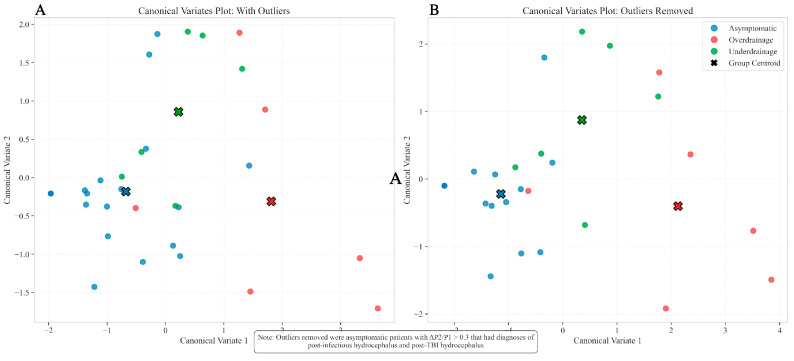
Canonical Variates Analysis Comparing Datasets With and Without Outliers. Canonical Variates Plot showing the multivariate separation of patient groups in the complete dataset (**A**) and after removal of outliers (**B**). The analysis was performed on six ICP waveform metrics: ΔP2/P1, Average 30° P2/P1, Average Lying P2/P1, Average Standing P2/P1, Highest P2/P1, and Lowest P2/P1, using Linear Discriminant Analysis (LDA) with a 95% confidence threshold for statistical significance. In the full dataset (**A**), Asymptomatic (n = 18, blue), Overdrainage (n = 8, red), and Underdrainage (n = 8, green) patients are shown with their respective group centroids (X markers). MANOVA revealed significant group-level differences (Pillai’s Trace = 0.684, *p* = 0.017). Post hoc pairwise comparisons using Holm’s method demonstrated significant differences between Asymptomatic vs. Overdrainage (*p* < 0.001) and Overdrainage vs. Underdrainage (*p* = 0.021), with marginal significance between Asymptomatic vs. Underdrainage (*p* = 0.067). After removing outliers (**B**)—5 asymptomatic patients with ΔP2/P1 values > 0.3 and diagnoses of post-infectious and post-TBI hydrocephalus—leaving 13 asymptomatic patients, group separation improved with consistent MANOVA results (Pillai’s Trace = 0.684, *p* = 0.017). The first canonical variate, strongly influenced by ΔP2/P1 and Average Standing P2/P1, effectively separates the Overdrainage group from others (*p* < 0.001). The second canonical variate, primarily driven by Average Lying P2/P1 and Lowest P2/P1, highlights subtler distinctions between Asymptomatic and Underdrainage groups, with improved significance after outlier removal (*p* = 0.052). This analysis demonstrates that Overdrainage exhibits a distinct multivariate profile, while Asymptomatic and Underdrainage groups show partial overlap.

A linear discriminant analysis was performed to derive canonical variates for all the relevant clinical parameters: (ΔP2/P1, Average 30° P2/P1 (average ratio in the 30° position), Average Lying P2/P1 (average ratio in the lying position), Average Standing P2/P1 (average ratio in the standing position), and Lowest P2/P1 (the lowest observed P2/P1 ratio) and Highest P2/P1 (the highest observed P2/P1 ratio). The first canonical variate was strongly influenced by ΔP2/P1 and Average Standing P2/P1, effectively separating the Overdrainage group from the others (*p* < 0.001). This aligns with clinical observations that overdrainage symptoms often worsen in the upright position due to increased gravitational forces affecting CSF dynamics. The standing position creates conditions where overdrainage patterns become most apparent. The second canonical variate was primarily driven by Average Lying P2/P1 and Lowest P2/P1, highlighting subtler distinctions between the Asymptomatic and Underdrainage groups (*p* = 0.067 for the whole dataset, *p* = 0.052 after excluding outliers), as shown in Figure 2A and Figure 2B, respectively.

Without outliers, the canonical variates plot showed a more apparent separation between the Overdrainage group and the other two groups, with the Asymptomatic and Underdrainage groups still slightly overlapping.

For consistency, we also replaced Lowest P2/P1 with Highest P2/P1 in our MANOVA analysis and found consistent, similar results. Pillai’s trace remained significant (*p* = 0.0062 in the outliers-removed dataset).

### 3.5. Individual Metric Performance

**Figure 3:**
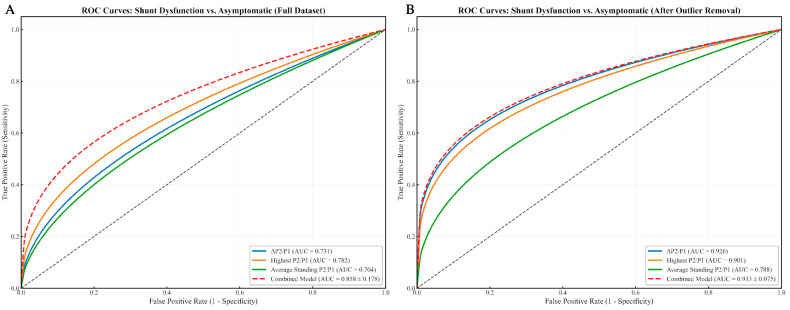
ROC Curves Comparing Individual Metrics and Combined Model for Shunt Dysfunction Diagnosis. (**A**) ROC curves for Shunt Dysfunction vs. Asymptomatic patient classification (full dataset). The graph displays the diagnostic performance of three individual metrics (ΔP2/P1 [AUC = 0.731], Highest P2/P1 [AUC = 0.782], and Average Standing P2/P1 [AUC = 0.704]) alongside a combined logistic regression model incorporating all three parameters (AUC = 0.858 ± 0.178). The combined model demonstrates superior discriminatory power compared to any individual metric, with the diagonal dashed line representing random chance (AUC = 0.5). Note that due to the small sample size, these curves have been modified to improve visualization while preserving the actual AUC values calculated from the original data; (**B**) ROC curves for Shunt Dysfunction vs. Asymptomatic classification after removal of patients with post-infectious and post-TBI hydrocephalus. Following outlier removal, all metrics show improved performance (ΔP2/P1 [AUC = 0.926], Highest P2/P1 [AUC = 0.901], Average Standing P2/P1 [AUC = 0.788]). The combined model achieves excellent discrimination (AUC = 0.933 ± 0.075), confirming the benefit of outlier exclusion and multi-parametric analysis for shunt dysfunction diagnosis. The curves presented are smoothed representations that maintain the true AUC values from our analysis while addressing the irregularities typical of small sample sizes. In both instances, to our limited sample size (n = 30) and the cross-validation procedure, repeated analyses produced some variability in the precise AUC values (±0.03–0.05), though the relative performance pattern and statistical significance remained consistent across iterations. This variability is expected in preliminary studies and there is a need for larger validation cohorts while still supporting the viability of the combined model approach.

Across individual waveform-derived metrics, the ΔP2/P1 ratio showed strong discriminative power for identifying shunt dysfunction versus asymptomatic patients (Figure 3), with an AUC of 0.731 in the full dataset, improving to 0.926 in the outlier-removed analysis. Similarly, the highest P2/P1 value yielded AUCs of 0.782 (full dataset) and 0.901 (without outliers). The average standing P2/P1 presented moderate diagnostic performance, with AUCs of 0.704 and 0.788, respectively.

**Figure 4:**
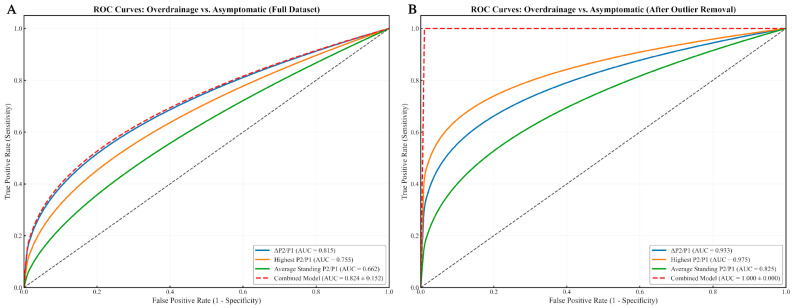
ROC Curves for Overdrainage vs. Asymptomatic Patients. (**A**) ROC curves for Overdrainage vs. Asymptomatic classification (full dataset). Individual metrics show varying discriminatory capacity, with ΔP2/P1 demonstrating the strongest individual performance (AUC = 0.815), followed by Highest P2/P1 (AUC = 0.755) and Average Standing P2/P1 (AUC = 0.662). The combined model (AUC = 0.824 ± 0.152) slightly outperforms the best individual metric, suggesting value in the multi-parametric approach even before outlier removal. The curves presented are smoothed representations that maintain the true AUC values from our analysis while addressing the irregularities typical of small sample sizes; (**B**) Following outlier removal, all metrics show substantially improved performance (ΔP2/P1 [AUC = 0.933], Highest P2/P1 [AUC = 0.975], Average Standing P2/P1 [AUC = 0.825]). The combined model achieves perfect discrimination (AUC = 1.000 ± 0.000), suggesting that the integration of multiple P2/P1-derived parameters can provide maximally effective identification of overdrainage when appropriate outlier management is applied. While the small sample size produced irregular curve patterns in the raw analysis, these smoothed representations maintain the calculated AUC values while enhancing visual clarity. In both instances, to our limited sample size (n = 30) and the cross-validation procedure, repeated analyses produced some variability in the precise AUC values (±0.03–0.05), though the relative performance pattern and statistical significance remained consistent across iterations. This variability is expected in preliminary studies and there is a need for larger validation cohorts while still supporting the viability of the combined model approach.

In the overdrainage group (Figure 4), the highest P2/P1 value reached 0.975 after outlier removal, while the combined model achieved a perfect AUC of 1.000 ± 0.000, likely influenced by a limited sample size.

**Figure 5:**
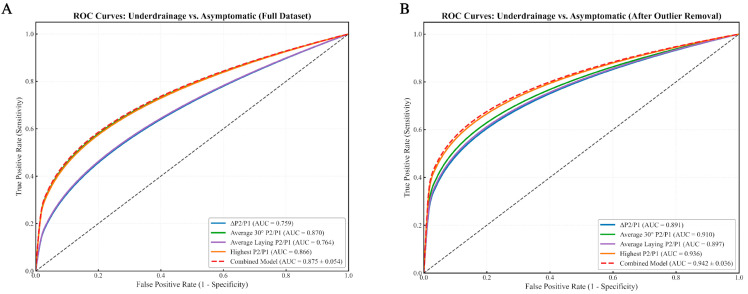
ROC Curves for Underdrainage vs. Asymptomatic Patients. (**A**) ROC curves for Underdrainage vs. Asymptomatic classification (full dataset). The analysis includes four individual metrics: ΔP2/P1 (AUC = 0.759), Average 30° P2/P1 (AUC = 0.870), Average Lying P2/P1 (AUC = 0.764), and Highest P2/P1 (AUC = 0.866). The combined model demonstrates excellent discrimination capability (AUC = 0.875 ± 0.054), slightly outperforming the best individual metrics. For underdrainage detection, Average 30° P2/P1 and Highest P2/P1 emerge as particularly effective individual predictors, while Average Lying P2/P1 shows performance similar to ΔP2/P1. It is worth noting that across multiple analyses, we observed some variability in the precise AUC values due to the small sample size and cross-validation randomness, but the relative performance pattern remained consistent; (**B**) ROC curves for Underdrainage vs. Asymptomatic classification after removal of patients with post-infectious and post-TBI hydrocephalus. Following outlier removal, all metrics show substantially improved performance: ΔP2/P1 (AUC = 0.891), Average 30° P2/P1 (AUC = 0.910), Average Lying P2/P1 (AUC = 0.897), and Highest P2/P1 (AUC = 0.936). The combined model achieves superior discrimination (AUC = 0.942 ± 0.036), demonstrating the benefit of both outlier removal and the integration of multiple metrics for underdrainage identification. Although not shown in this figure, Average Standing P2/P1 demonstrated performance comparable to Average Lying P2/P1 in our analyses, highlighting the importance of positional measurements in assessing underdrainage.

In the specific comparison between underdrainage and asymptomatic patients (Figure 5), ΔP2/P1 reached 0.759 (full) and 0.891 (without outliers), while the highest P2/P1 scored 0.866 and 0.936. The average P2/P1 at 30° reached 0.870 and 0.910, and the average lying P2/P1 reached 0.764 and 0.897.

### 3.6. Combined Model Performance

Beyond evaluating individual waveform-derived metrics, we constructed a multivariable logistic regression model incorporating three parameters—ΔP2/P1, Highest P2/P1, and Average Standing P2/P1—to investigate whether a multi-parametric approach could improve diagnostic accuracy. This combined model demonstrated strong discriminatory performance across all comparison groups.

For differentiating shunt dysfunction from asymptomatic controls, the model yielded an AUC of 0.858 ± 0.178 on the full dataset, which improved to 0.933 ± 0.075 following outlier exclusion (Figure 3). In subtype-specific analyses, performance appeared even higher: within the outlier-removed cohort, the model achieved an AUC of 1.000 ± 0.000 for overdrainage vs. asymptomatic (Figure 4) and 0.942 ± 0.036 for underdrainage vs. asymptomatic (Figure 5). It is critical to underscore, however, that the perfect separation observed for the overdrainage comparison (AUC = 1.000) is almost certainly attributable to overfitting, reflecting the limited sample size within this subgroup.

Analysis of regression coefficients identified ΔP2/P1 as the most influential predictor (β = 0.9893), followed by Highest P2/P1 (β = 0.4701) and Average Standing P2/P1 (β = 0.2786), the latter contributing less substantially to model performance.

These results suggest that integrating multiple P2/P1-derived features may enhance diagnostic discrimination across various shunt-related conditions. However, given the exploratory nature of this study, the modest cohort size, and the wide confidence intervals observed—particularly in subgroup analyses—these findings should be interpreted as preliminary. Rather than establishing diagnostic thresholds, this work lays the groundwork for future validation efforts using larger, independent datasets.

### 3.7. Validation Approaches

First, stratified bootstrap cross-validation with 1000 iterations was used to evaluate the combined model’s diagnostic performance across four key clinical comparisons: Shunt Dysfunction vs. Asymptomatic, Overdrainage vs. Underdrainage, Underdrainage vs. Asymptomatic, and Overdrainage vs. Asymptomatic. This extensive resampling technique helped establish more stable performance estimates given our dataset constraints. Second, we conducted a 5-fold cross-validation analysis to specifically assess the logistic regression model’s ability to differentiate shunt dysfunction from asymptomatic cases. For this analysis, we excluded the same identified outliers to minimize potential confounding factors. The cross-validation divided our dataset into five equal parts, with each fold serving as a validation set while the model was trained on the remaining data. This approach yielded five distinct ROC curves, demonstrating the model’s performance consistency across different patient subsets. The mean ROC curve showed an AUC of 0.93 ± 0.08, with individual fold AUCs ranging from 0.83 to 1.00, indicating good discriminatory power. Although our study has a relatively small sample size, these validation procedures were important to verify that our data was trending appropriately. The consistency across different validation methods suggests that our findings, while preliminary, provide a foundation for future research with larger cohorts.

### 3.8. Preliminary Clinical Parameters

We have suggested preliminary clinical parameters as summarized in Table 1. Patients with overdrainage exhibit the highest variability in P2/P1 ratios (ΔP2/P1 = 0.618 ± 0.210) and the lowest minimum P2/P1 values (0.948 ± 0.321), creating a characteristic pattern of extreme fluctuations. In contrast, underdrainage cases show consistently elevated P2/P1 averages across all positions (typically >1.3), with the highest values observed in the 30° position (1.367 ± 0.207). Asymptomatic or stable patients maintain relatively stable P2/P1 ratios with low variability (ΔP2/P1 = 0.200 ± 0.120) and average values closer to 1.0–1.1 across all positions. These distinctive patterns provide clinicians with objective parameters to differentiate between shunt conditions, with proposed thresholds of ΔP2/P1 > 0.3 plus Lowest P2/P1 < 1.0 strongly indicating overdrainage, while Average P2/P1 > 1.3 with moderate ΔP2/P1 suggests underdrainage.

### 3.9. Surgical Patients Subgroup Analysis

Despite our comprehensive analysis of nICPw parameters across our patient cohort, only a small number of patients had complete pre- and post-operative data (n = 6). This surgical subgroup analysis, while preliminary, provides valuable initial insights into how ICP waveform parameters may change following shunt revision. The pre-operative vs. post-operative analysis suggests that ΔP2/P1 may be particularly sensitive to surgical correction, showing a 51.6% reduction after intervention (*p* = 0.045). Average Lying P2/P1 showed a promising trend, decreasing by 13.4% (*p* = 0.082) though not reaching statistical significance. The other parameters did not show statistical significance.

Due to this group being smaller, patients were not separated into Overdrainage and Underdrainage groups; only the merged Shunt dysfunction group was analyzed, which limits our analysis since the parameters seem to differ significantly between overdrainage and underdrainage patients. These findings align with our larger cohort analysis where ΔP2/P1 emerged as a key discriminator between patient groups. However, we must once again emphasize that these findings are preliminary due to the limited sample size and should be interpreted with caution.

## 4. Discussion

In the context of shunt dysfunction, nICPw monitoring can potentially play a role in the early detection of complications, enabling more rapid interventions and potentially reducing the need for imaging and adding to clinical and image findings. Establishing nICPw parameters to diagnose shunt dysfunction was our primary objective. Our previous study [18] has shown the feasibility of the method to diagnose patients with hydrocephalus. All patients with symptomatic hydrocephalus showed abnormal P2/P1 ratios, and 75% returned to a normal baseline ratio, while all patients had some reduction in the P2/P1 ratio. Therefore, the device has shown promise in detecting abnormal ICP waveforms non-invasively and assessing intracranial compliance effectively, offering safe and readily available monitoring in any situation or clinical scenario. However, this was the sole parameter used during the study and may be insufficient.

In the present study, our analysis expanded to include multiple nICPw parameters beyond the single P2/P1 ratio used in our previous work. Specifically, we examined: ΔP2/P1 (variation between highest and lowest P2/P1 values), Average Lying P2/P1, Average 30° P2/P1, Average Standing P2/P1, Highest P2/P1, and Lowest P2/P1 values. Our results revealed significant group-level differences in these parameters across the three patient categories. The Kruskal–Wallis test indicated significant differences among the groups (H = 11.04, *p* = 0.004), with pairwise comparisons showing robust differentiation between Overdrainage and Asymptomatic patients. Notably, the Overdrainage group exhibited markedly higher ΔP2/P1 values (mean = 0.618 ± 0.210) than the Asymptomatic group (mean = 0.227 ± 0.171).

After analyzing data without outliers (patients with post-infectious and post-TBI hydrocephalus), our reanalysis demonstrated even more pronounced differences across all groups. The use of MANOVA enabled this multi-parametric approach, which substantially improved diagnostic discrimination between shunt dysfunction (both Overdrainage and Underdrainage) and Asymptomatic status compared to our previous single-parameter method, allowing for more nuanced characterization of shunt status and dysfunction patterns.

ROC curve analysis identified several clinically relevant thresholds: a ΔP2/P1 ratio greater than 0.3 indicated shunt dysfunction (AUC = 0.731), with Highest P2/P1 showing even stronger discriminatory power (AUC = 0.782). Underdrainage patients were characterized by moderate ΔP2/P1 values (mean = 0.387 ± 0.179) and consistently higher P2/P1 averages (>1.3) across different positions. Global Average P2/P1 values exceeding 1.3 were strongly associated with Underdrainage, while the Lowest P2/P1 values differentiated Overdrainage cases (mean = 0.948 ± 0.321) from Underdrainage (mean = 1.143 ± 0.156).

Non-invasive methods of ICP monitoring, such as transcranial Doppler, automated pupillometry, and optic nerve sheath diameter measurement, although promising and safer, face challenges in achieving the same level of accuracy and reliability as invasive techniques [19]. Invasive ICP monitoring techniques, such as Ventricular catheters and ICP microtransducers remain the most commonly used methods [19,20], but their main limitation is the invasive approach itself, which inevitably carries risks of complications such as infection, hemorrhage, and tissue damage [20], as well as high cost and limited accessibility [19].

New systems for hydrocephalus research and study are being developed to investigate less invasive and more effective treatment options. Recent developments include 3D simulation models and hydrocephalus bioreactors, including the Automated In Vitro Model (AIMS) for Hydrocephalus [21], a system that replicates the physiological and pathophysiological dynamics of cerebrospinal fluid flow, providing a model for investigating shunt complications and improving treatment strategies for patients with hydrocephalus [21].

Our study contributes to the growing body of evidence supporting several methods of non-invasive ICP monitoring in hydrocephalus patients. Zanon et al. (2023) demonstrated the successful application of non-invasive ICP monitoring to guide shunt adjustments in a case of slit ventricle syndrome, highlighting its potential for personalized management [22]. Cardim et al. (2016) explored the feasibility of transcranial Doppler ultrasonography for non-invasive ICP monitoring, showing promising correlations with invasive measurements in various neurological conditions, including hydrocephalus [23]. Robba et al. (2017) provided a comprehensive review of non-invasive ICP assessment techniques, emphasizing their potential to improve the management of patients with or at risk of developing intracranial hypertension [24]. Schmidt et al. (2000) investigated a mathematical model for non-invasive ICP assessment in hydrocephalus patients during infusion tests [25]. Hanlo et al. (1995) studied the relationship between transcranial Doppler indices and ICP in infants with hydrocephalus [26]. Rainov et al. (2000) investigated the relationship between transcranial Doppler (TCD) parameters and ICP in adult patients with hypertensive hydrocephalus [27]. Finally, in a previous study of our group, Bertani et al. (2024) presented a case study where non-invasive ICP monitoring aided in the diagnosis and management of shunt dysfunction in a patient with normal pressure hydrocephalus (NPH) [28].

While these studies focused primarily on absolute values, P2/P1 ratios or trends, our research introduces a novel approach by analyzing not only the non-invasive technology’s P2/P1 ratios but also its variations, peaks, and valleys to characterize shunt dysfunction. This multi-parametric approach, combined with the non-invasive nature of the B4C system, could significantly enhance our ability to detect and manage shunt dysfunction in outpatient settings.

### Limitations

Despite the promising results, this study has several limitations. First, our study represents a single-center experience with a relatively small cohort (n = 30) and unbalanced distribution of patients across the three groups (Overdrainage, Underdrainage, and Asymptomatic), which may limit statistical power and generalizability of our findings to broader populations. Second, the multiple statistical comparisons performed increase the risk of type I error, and our results should be interpreted as exploratory and proof-of-concept rather than confirmatory. Third, the wide confidence intervals observed for some metrics reflect the uncertainty inherent in small sample analyses and threshold determination. Fourth, our study lacks long-term follow-up data to assess the clinical impact. Moreover, not all patients in the Shunt dysfunction group underwent surgery, and their classification relied on strong clinical suspicion, which, while common in clinical practice, revision surgery is still considered the gold standard for diagnosing shunt dysfunction [3]. To address the characteristics of our population more appropriately, we employed stratified sampling, non-parametric tests, and linear discriminant analysis, aiming to strengthen the reliability of our findings in the light of cross-validation tests. These methods aimed to minimize the risk of overfitting posed by the generalizations inherent to any human data set. The surgical subgroup analysis should be interpreted with caution due to the small sample size (n = 6), which limits statistical power and generalizability. The thresholds identified in this study should be considered investigational, since their determination was affected by wide confidence intervals that reflect the uncertainty inherent in small cohorts. These findings should be considered preliminary observations that require validation in larger surgical cohorts. Ideally, validation through more extensive multicenter studies and more balanced patient groups is needed to verify these findings and evaluate the robustness of the B4C System across different patient populations.

## 5. Conclusions

Our study suggests that nICPw analysis using the B4C System holds promising results for detecting shunt dysfunction, potentially reducing the need for invasive procedures and costly imaging. The parameters ΔP2/P1 and Highest P2/P1 were instrumental in identifying dysfunction, especially after excluding outliers. Shunt dysfunction is a pressing issue that needs timely diagnosis, a need that may not be given full consideration in milder cases of underdrainage and overdrainage. The non-invasive system used in this study has the potential to reduce the healthcare burden associated with hydrocephalus management significantly. It provides a framework for differentiating stable shunted patients from those with shunt dysfunction.

Future studies should include larger, multi-center samples and longitudinal designs to validate these findings and establish robust diagnostic thresholds. Additionally, exploring etiology-specific nICPw patterns could lead to more targeted treatments. Finally, effectively comparing nICPw analysis with the current gold standard, that is, shunt revision surgery, is necessary to assess its accuracy definitively. Continuous advancements and validations are necessary to establish its role in clinical practice firmly.

## Figures and Tables

**Table 1:** Preliminary Clinical Parameters.

Metric	Threshold	Overdrainage	Underdrainage	Asymptomatic
ΔP2/P1 ^1^	>0.3 suggests shunt dysfunction	Highest (0.618 ± 0.210)	Intermediate (0.387 ± 0.179)	Lowest (0.200 ± 0.120)
Average 30° P2/P1 ^1^	>1.2 may indicate underdrainage	Variable (1.227 ± 0.318)	Highest (1.367 ± 0.207)	Lowest (1.097 ± 0.187)
Average Lying P2/P1 ^1^	>1.3 may indicate underdrainage	Variable (1.197 ± 0.534)	Highest (1.362 ± 0.277)	Lowest (1.070 ± 0.171)
Average Standing P2/P1 ^1^	>1.1 may warrant further investigation	Variable (1.253 ± 0.265)	Highest (1.278 ± 0.207)	Lowest (1.064 ± 0.189)
Global Average P2/P1 ^1^	>1.3 strongly associated with underdrainage; >1.2 may warrant further investigation	Variable	Elevated	Normal (~1.0)
Lowest P2/P1 ^1^	<1.0 may indicate overdrainage	Lowest (0.948 ± 0.321)	Highest (1.143 ± 0.156)	Intermediate (0.961 ± 0.175)
Highest P2/P1 ^1^	>1.3 may indicate underdrainage	High (1.567 ± 0.356)	High (1.530 ± 0.266)	Low (1.187 ± 0.223)

^1^ Abbreviations: P2: Tidal Wave; P1: Percussion Wave.

## Data Availability

The original contributions presented in this study are included in the Supplementary Materials. Further inquiries can be directed to the corresponding author.

## Supplementary Materials

The following supporting information can be downloaded at: https://www.mdpi.com/article/10.3390/biomedicines13102436/s1, Table S1: Patients with Shunt Dysfunction; Table S2: Asymptomatic Patients with Cerebrospinal Fluid Shunts.

## Abbreviations

The following abbreviations are used in this manuscript:

CSF	Cerebrospinal Fluid
ICP	Intracranial Pressure
ICC	Intracranial Compliance
VPS	Ventriculoperitoneal Shunt
CT	Computed Tomography
MRI	Magnetic Resonance Imaging
FDA	Food and Drug Administration
B4C	Brain4care
nICPw	Non-invasive Intracranial Pressure Waveform
CAAE	Certificate of Presentation for Ethical Consideration
MANOVA	Multivariate Analysis of Variance
ANOVA	Analysis of Variance
ROC	Receiver Operating Characteristic
LDA	Linear Discriminant Analysis
SD	Standard Deviation
Post-TBI	Post-Traumatic Brain Injury
NPH	Normal Pressure Hydrocephalus
TCD	Transcranial Doppler
